# Extraction and Characterization of Cellulose from Agricultural By-Products of Chiang Rai Province, Thailand

**DOI:** 10.3390/polym14091830

**Published:** 2022-04-29

**Authors:** Orapan Romruen, Thomas Karbowiak, Wirongrong Tongdeesoontorn, Khursheed Ahmad Shiekh, Saroat Rawdkuen

**Affiliations:** 1 Food Science and Technology Program, School of Agro-Industry, Mae Fah Luang University, Chiang Rai 57100, Thailand; orapan.rom13@lamduan.mfu.ac.th; 2 UMR PAM-Food and Wine Science & Technology, Agro-Sup Dijon, Université de Bourgogne France-Comte, Esplanade Erasme, F-21000 Dijon, France; thomas.karbowiak@agrosupdijon.fr; 3 Unit of Innovative Food Packaging and Biomaterials, School of Agro-Industry, Mae Fah Luang University, Chiang Rai 57100, Thailand; wirongrong.ton@mfu.ac.th (W.T.); khursheed.research@mfu.ac.th (K.A.S.)

**Keywords:** agricultural waste valorization, alkaline extraction, cellulose, FTIR, XRD, thermal properties, biodegradable, packaging

## Abstract

Cellulose is an abundant component of the plant biomass in agricultural waste valorization that may be exploited to mitigate the excessive use of synthetic non-biodegradable materials. This work aimed to investigate the cellulose utilized by alkaline extraction with a prior bleaching process from rice straw, corncob, Phulae pineapple leaves, and Phulae pineapple peels. The bleaching and alkaline extraction process was performed using 1.4% acidified sodium chlorite (NaClO_2_) and 5% potassium hydroxide (KOH) in all the samples. All the samples, without and with the alkaline process, were characterized for their physico-chemical, microstructure, thermal properties and compared to commercial cellulose (COM-C). The extraction yield was the highest in alkaline-extracted cellulose from the corncob (AE-CCC) sample (*p* < 0.05), compared to the other alkaline-treated samples. The undesired components, including mineral, lignin, and hemicellulose, were lowest in the AE-CCC sample (*p* < 0.05), compared to raw and alkaline-treated samples. The microstructure displayed the flaky AE-CCC structure that showed a similar visibility in terms of morphology with that of the alkaline-treated pineapple peel cellulose (AE-PPC) and COM-C samples compared to other alkaline-treated samples with a fibrous structure. Fourier Transform Infrared (FTIR) and X-ray Diffraction (XRD) of AE-CCC samples showed the lowest amorphous regions, possibly due to the elimination of hemicellulose and lignin during bleaching and alkaline treatment. The highest crystallinity index obtained in the AE-CCC sample showed a close resemblance with the COM-C sample. Additionally, the AE-CCC sample showed the highest thermal stability, as evidenced by its higher T_onset_ (334.64 °C), and T_max_ (364.67 °C) compared to the COM-C and alkaline-treated samples. Therefore, agricultural wastes after harvesting in the Chiang Rai province of Thailand may be subjected to an alkaline process with a prior bleaching process to yield a higher cellulose content that is free of impurities. Thus, the extracted cellulose could be used as an efficient, eco-friendly, and biodegradable material for packaging applications.

## 1. Introduction

Synthetic materials are employed to produce the packaging materials obtained from petroleum-based by-products that, after a single use, are disposed of in the environment [1]. The application of synthetic and non-biodegradable polymer-tailored materials for the packaging of commodities has raised alarming global consequences that draw researchers’ attention to the investigation of bio-based and eco-friendly sources [2]. At present, there is a growing demand for biodegradable materials that are ecologically compatible, which are mostly derived from the renewable resources of agricultural wastes.

The agriculture sector accounts for about 46% of the total land production area in Thailand [3]. As a result, fresh fruits, and cereals, such as different cultivars of rice export quality, are grown in crop-friendly climatic conditions. The exportation record for rice from the year 2021 was estimated to be 4.6 million tons [3]. Apart from rice cultivation, several economic crops, such as corn, cassava, palm oil, rubber, sugarcane, and tropical fruits, largely boost Thailand’s economic growth. In addition, corn cultivation has been an essential source of 80–100% of the processed products for animal feed and human consumption [4]. The production of rice and corn cultivation at 0.90 and 0.23 million tons in 2021 has been documented in Chiang Rai, a northern province of Thailand [3]. Fresh fruits serve as a backbone for farmers in the rural areas of Chiang Rai, Thailand. Fresh fruits such as pineapples, oranges, mangoes, and bananas are grown in the northern part of Thailand. A cultivar of pineapple (*Ananas comosus* L.) Merr., popularly known as “Phulae” can be only grown in certain parts due to its compatibility with the geographical location and climatic conditions of Chiang Rai, Thailand [5]. The Phulae variety of pineapple has a high demand as a fresh-cut product that is exported to different countries [6].

Increased agricultural production generates a bulk quantity of agricultural wastes after crop harvesting in the fields. A significant quantity of these wastes remains underutilized in the form of rice straw, pineapple leaves, and corn cobs in the harvesting fields of northern Thailand. Eventually, most of these wastes are burnt by the local farmers in an open space, generating a large quantity of smoke that severely impacts human health and the environment. Most farmers are unaware of the value of waste recycling and its economic potential due to lack of knowledge [7]. The majority of agricultural wastes are composed of lignocellulosic materials, specifically cellulose, hemicellulose, and lignin [8]. The production of lignocellulosic materials such as cellulose into value-added products has attracted considerable attention among academics and industry players. It is often regarded as a highly adaptable resource for the replacement of petroleum-based materials due to its ease of access, stability, low density, non-abrasive, non-toxic, renewable, and biodegradable nature [9]. Cellulose is the most abundant biopolymer in plants [10]. Cellulose may be produced from plant sources in the form of whisker-like fibrils with a linear homopolymer chain of beta-D,1, 4 glucose units linked by glycosidic bonds [11,12]. Cellulose has several advantages in its use as a reinforcement material with low density and high mechanical properties to develop biopolymer composites [13,14].

The extraction of cellulose from agricultural wastes is subjected to pre-treatments using toluene, ethanol, or petroleum ether to remove the lipids, wax, phenolic, and pigments [15,16]. After the removal of impurities, an alkaline process has been recommended, such as sodium hydroxide (NaOH), potassium hydroxide (KOH), and organic solvents [17,18,19]. The alkaline process involves the hydrolysis of cellulose polymers to enhance performance by exposing the interior surface structure of the fiber in rapeseed hulls [17]. Alkaline hydrolysis is followed by the bleaching process of cellulose fibers using sodium chlorite (NaClO_2_) and hydrogen peroxide (H_2_O_2_) to remove all the residual lignin, wax, and lipids [17]. However, the cellulose-rich by-products rice straw (RS), corncob (CC), Phulae pineapple leaf (PL), and Phulae pineapple peel (PP) were considered in this study because the Phulae pineapple fresh-cut industries, and the production of rice and corn, are expected to generate even greater amounts in the province. Therefore, the cellulose of the aforementioned by-products was extracted through a bleaching and alkaline process with the same conditions. Furthermore, various techniques were used to characterize the extracted celluloses. The size, shape, and morphology of extracted cellulose were observed using Scanning Electron Microscopy (SEM). Changes in chemical behaviors and crystallinity were analyzed by Fourier transform infrared (FTIR) and X-ray diffraction (XRD). The thermal stability of the extracted cellulose was analyzed using a thermogravimetric analysis (TGA) instrument. All the measured properties were compared with commercial cellulose.

## 2. Materials and Methods

### 2.1. Materials

Commercial cellulose from cotton was purchased from Chanjao Longevity Co., Ltd. (Bangkok, Thailand). Potassium hydroxide (KOH, 85%) was procured from Sigma Aldrich Co. (St Louis, MO, USA). Toluene (99.8%) and ethanol (C_2_H_6_O, 95%) were attained from RCI Labscan Co., Ltd. (Bangkok, Thailand). Sodium chlorite (NaClO_2_, 80%) was bought from Ajax Finechem Pty Ltd. (Scoresby VIC 3179, Australia). Acetic acid glacial (CH_3_COOH, 99.7%) was purchased from QRëC™ (Auckland, New Zealand). All the materials and chemicals used in our study were of analytical grade.

### 2.2. Preparation and Extraction of Cellulose from Agricultural By-Products

Rice straws (RS) (*Oryza sativa* var. glutinosa), corncob (CC) (*Zea mays* var. indentata), Phulae pineapple leaves (PL) (*Ananas comosus* var. Phulae), and Phulae pineapple peels (PP) (*Ananas comosus* var. Phulae) were collected from the agricultural fields of Thasud, Chiang Rai, Thailand in the month of September 2021. RS, CC, PL, and PP raw materials with no apparent damage were collected and transported to the Department of Food Technology, Mae Fah Luang University, Chiang Rai, Thailand. All the samples were washed with distilled water to remove dust and adhered soils particles. After the washing process, all the samples were cut into 3–5-cm pieces and dried in a hot air oven at 60 °C for 48 h. The dried samples were ground, and 30 g of each RS, CC, PL, and PP samples were dissolved in 450 mL of toluene: ethanol (2:1) and left for shaking in a closed cabinet of bench-top temperature-controlled orbital shaker (IKA KS 3000 i control, IKA-Werke GmbH & Co., Staufen, Germany) at 150 rpm and 25 °C for 20 h. All the samples collected after the shaking process were filtered through Whatman No. 4 filter paper (Schleicher and Schuell, Maidstone, England) using a Buchner funnel equipped with a vacuum pump. After removal of the solvent by filtration, all the samples were washed with absolute ethanol and filtered again before drying for 1 h at 100 °C. Dried fibrous matter of all the samples was mixed with 1.4% (*w*/*v*) sodium chlorite (NaClO_2_) to bleach the sample fibers. The pH of all the samples was adjusted to 4 by using 5% (*v*/*v*) acetic acid solution followed by heating at 70 °C with continuous stirring using an overhead stirrer at 500 rpm for 5 h. The sample fibers were filtered and washed with distilled water until a neutral pH was maintained and dried at 100 °C for 16 h. After bleaching, dried fibers were soaked in 600 mL of 5% (*w*/*v*) potassium hydroxide (KOH) and stirring was continued at 500 rpm under room temperature for 24 h prior to heating at 90 °C for 2 h for the extraction of cellulose. The KOH-treated samples were washed with distilled water until a neutral pH was attained, followed by drying in a hot-air oven at 100 °C for 20 h to obtain cellulose [20]. All the samples were subjected to physical, chemical, microstructural, and thermal analyses.

### 2.3. Physico-Chemical Composition Analysis of Cellulose from Agricultural Waste

The percent extraction yield of cellulose from RS, CC, PL and PP samples was calculated considering the mass of bagasse-extracted cellulose (g) and the dried mass of bagasse (g) using the Equation (1), as follows [21].

Yield (%) = (Weight of cellulose)/(Weight of dried bagasse) × 100 (1)

The chemical composition of all the cellulose fiber samples were determined according to the standard method of Technical Association of Pulp and Paper Industry standard (TAPPI). The lignin contents of all the samples were measured using T222 om-98 method, and holocellulose contents (α-cellulose and hemicellulose) were estimated using the acid-chlorite method [22]. In addition, the α-cellulose content was investigated using T203 om-88, and hemicellulose content was calculated by subtracting the α-cellulose content from the holocellulose content. Finally, the ash content was determined according to AOAC [23].

### 2.4. Scanning Electron Microscopy (SEM) of Cellulose

A field emission scanning electron microscope (FESEM) (TESCAN, model, MIRA, Czech Republic) was employed to examine the microstructure of extracted cellulose samples. The samples were mounted on double-sided carbon tape, vacuum-dried, sputtered with gold, then scanned at a 3 kV accelerating voltage and 5000× magnification [24].

### 2.5. Fourier Transform Infrared (FTIR)

FTIR spectra of raw materials and cellulose samples were analyzed using an FTIR spectrophotometer (PerkinElmer/FTIR spectrum GX, PerkinElmer, Waltham, MA, USA) to characterize the functional groups of the samples [25]. Samples were ground with KBr (1:100, *w*/*w*). The spectra were obtained in transmittance mode from a total scan of 32 scans with a resolution of 4 cm^−^^1^ over the 4000–400-cm^−^^1^ range.

### 2.6. X-ray Diffraction (XRD)

XRD patterns of fiber and cellulose were examined using an XRD diffractometer (PANalytical/X’ Pert Pro MPD, PANalytical, Almelo, The Netherlands). The machine was operated at 40 kV and 30 mA, equipped with Cu Kα radiation at a wavelength of 1.54056 Å using a nickel monochromator filtering wave. The samples were scanned at room temperature in a range of 2θ = 5–40° at a scanning rate of 0.4°/min. The percentage of crystallinity index (CI) was calculated following Equation (2):

CI (%) = ((I002 − Iam)/I002) × 100(2)

where I002 represents the peak intensity corresponding to the crystalline domain (2θ = 19.0°), and Iam represents the peak intensity corresponding to the crystalline domain (2θ = 22.6°).

### 2.7. Thermogravimetric Analysis (TGA) of Cellulose

The TGA of extracted cellulose was assessed through a thermogravimetric analyzer (Mettler Toledo/TGA/DSC3+ HT, Mettler Toledo, Greifensee, Switzerland), following the conditions of Rashid and Dutta [26]. Approximately 5 mg of the extracted cellulose from each raw material was subjected in N_2_ gas atmosphere, in which all the samples were heated from 25 °C to 600 °C at a heating rate of 20 °C/min and a gas flow rate of 60 mL/min. For TGA, Derivative Thermogravimetry (DTG) curves were used to calculate the onset (T_onset_) and maximum decomposition temperature (T_max_) of samples, while TGA curves were used to determine the char residue at 600 °C (%).

### 2.8. Statistical Analysis

Analysis of variance (ANOVA) and Duncan’s multiple range test were performed using a statistical program, SPSS (SPSS Inc., Chicago, IL, USA) version 26. Samples were analyzed at a significance level of *p* < 0.05. Three replications were carried out for extraction yield and Chemical composition and one replication for SEM, FTIR, XRD and thermal properties.

## 3. Results and Discussion

### 3.1. Physico-Chemical Composition of Cellulose Extracted without and with Alkaline Treatment from Agricultural By-Products

The extraction yield and chemical composition of different types of raw material and extracted cellulose samples are presented in Table 1. The extraction yield of cellulose extracted via alkaline method from RS, CC, PL, and PP samples was 32.26, 38.18, 16.60, and 9.05% (*w*/*w*), respectively. The highest extraction yield was attained in alkaline-extracted cellulose from corncob (AE-CCC) compared to the other samples with alkaline treatment (*p* < 0.05). The extraction yield was related to the α-cellulose content in raw materials. The ash content of alkaline-treated samples was lower than that of raw material samples (*p* < 0.05). The lower ash content might be due to the removal of minerals during bleaching and repeated washing cycles throughout the extraction process. The chemical components obtained in RS, CC, PL and PP samples were in the range of 33.18–45.81% for α-cellulose, 27.88–44.15% for hemicellulose, and 12.70–27.25% for lignin. In comparison to all the raw material samples, the CC sample showed the highest α-cellulose content (45.81%) (*p* < 0.05), followed by RS (45.45%), PL (35.35%), and PP (33.18%), respectively. The results were in line with the α-cellulose content of straw from cereal crops and fresh fruit by-product residues [27,28,29]. The impurities including lignin and hemicellulose were relatively lowered in the samples with alkaline treatment compared to the raw material samples (*p* < 0.05). Due to the removal of the impurities component, agriculture waste valorization of AE-RSC, AE-CCC, AE-PLC and AE-PPC samples reported that higher cellulose concentrations, ranging from 78.07 to 82.69% (*w*/*w*), were attained in alkaline-treated samples. The increase in the cellulose content was reported in agricultural waste samples subjected to alkaline-assisted extraction after the elimination of lignin and hemicellulose impurities [30]. The findings demonstrated that the type and chemical composition of the raw fiber samples were influenced by the bleaching process and alkaline treatment to yield higher cellulose content. In addition, four different raw materials yielded similar results in terms of composition with different numbers. These results are interesting because the user may have the freedom to process rice, pineapple, or corncob with the same machinery for industrial applications.

### 3.2. Microstructure of Cellulose Extracted from Agricultural By-Product Using Alkaline Process

**Figure 1:**
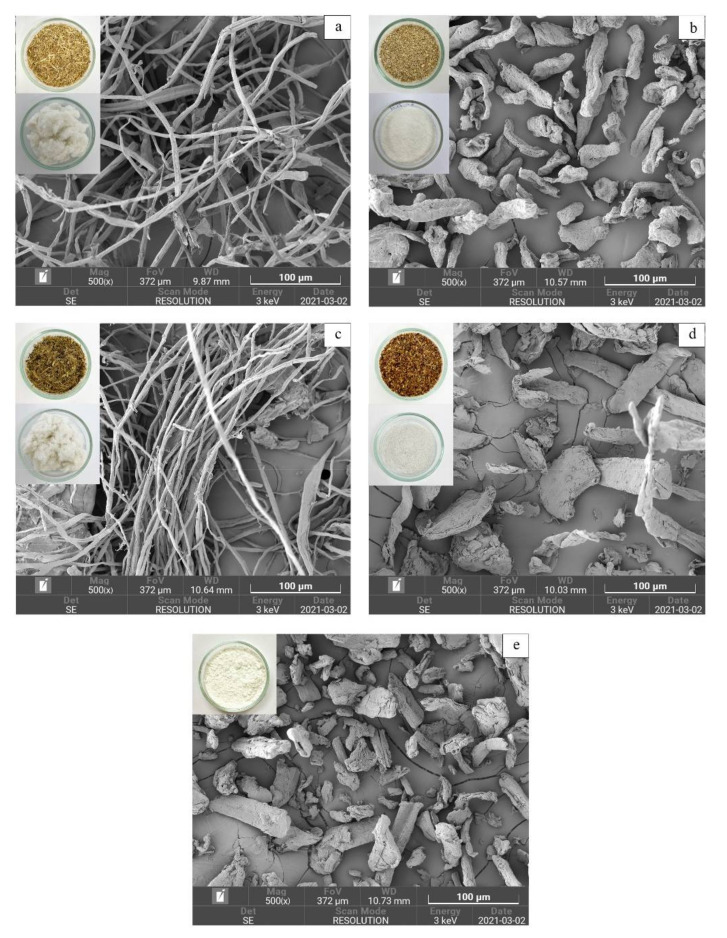
Scanning electron micrographs of cellulose samples with the alkaline processing of agricultural waste materials and their comparison with the commercial cellulose. (**a**) AE-RSC: Alkaline extraction of rice straw cellulose; (**b**) AE-CCC: Alkaline extraction of corncob cellulose; (**c**) AE-PLC: Alkaline extraction of pineapple leaf cellulose; (**d**) AE-PPC: Alkaline extraction of pineapple peels cellulose; and (**e**) COM-C: Commercial cellulose.

The appearance and microstructural morphology of AE-RSC, AE-CCC, AE-PLC, AE-PPC, and COM-C samples are displayed in Figure 1. The original colors of the raw material were brown, yellow–brown and green–brown. The cellulose is known as white in color. After bleaching and alkaline processing, the color of all extracted cellulose turned to white, which indicates that the major component is cellulose. The whiteness of the cellulose was attributed to the alkaline and bleaching treatment due to lignin and hemicellulose elimination [20]. Different shapes of cellulose samples were visualized using field emission scanning electron microscopy (FESEM). It was noted that AE-RSC and AE-PLC showed a whitish appearance, along with the fibrous geometry of cellulose fibrils (Figure 1a,c). On the other hand, the AE-CCC, AE-PPC, and COM-C exhibited a flaky structure with a rough surface and shorter fibril size (Figure 1b,d,e). The roughness and flaky appearance of cellulose might be due to defibrillation and the elimination of wax and other impurities [31]. The diameter of AE-RSC, AE-CCC, AE-PLC, AE-PPC, and COM-C samples was in the range of 3.03–22.00, 10.25–25.37, 2.76–10.44, 10.45–54.29, and 8.02–38.91 μm, respectively. In addition, the microstructure of the COM-C sample was correlated with the AE-CCC and AE-PPC samples, which revealed an identical fibril structure compared to the AE-RSC and AE-PLC samples. Moreover, the size of flake-shaped cellulose fibrils in AE-CCC was smaller, and the size of AE-PPC samples was comparatively larger than the COM-C sample. However, the size of whisker-like fibrils in AE-RSC and AE-PLC was relatively lower amongst all the samples. Cellulose samples with flaky appearances were dried to obtain the powdered form, while the whisker-shaped samples were more fibrous, to form a loop. Therefore, the cellulose attained in AE-CCC displayed a similar microstructure to that of commercial cellulose and could be used as another commercial source of cellulose in the market.

### 3.3. Fourier Transform Infrared (FTIR) of Cellulose Samples Extracted without and with Alkaline Process

**Figure 2:**
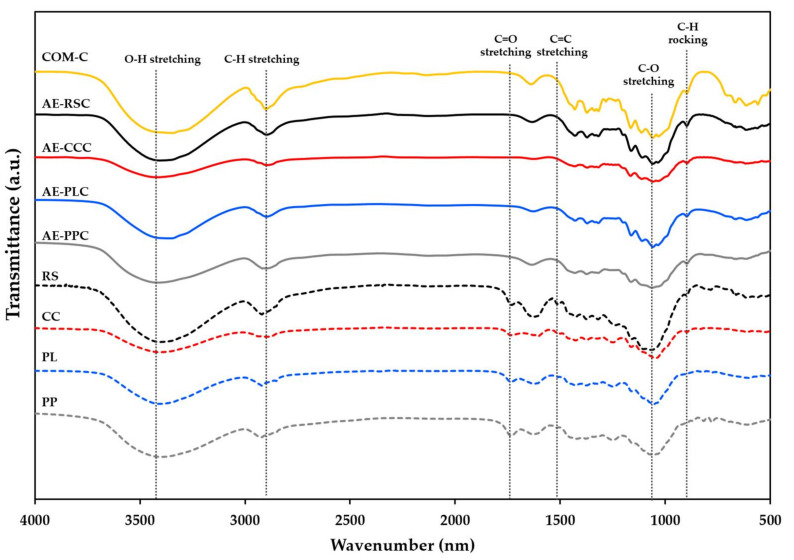
FTIR spectra of cellulose samples without and with alkaline processing of agricultural waste materials and their comparison with the commercial cellulose. RS: rice straw; CC: corncob; PL: pineapple leaf; PP: pineapple peel; AE-RSC: Alkaline extraction of rice straw cellulose; AE-CCC: Alkaline extraction of corncob cellulose; AE-PLC: Alkaline extraction of pineapple leaf cellulose; AE-PPC: Alkaline extraction of pineapple peels cellulose; and COM-C: Commercial cellulose.

FTIR analysis is a valuable method for studying the structural and physicochemical characteristics of polysaccharides. The FTIR spectra of different raw materials (without alkaline treatment) and extracted cellulose samples using the alkaline process are given in Figure 2. A dominant band was observed around 3400 cm^−1^ in all the raw materials, alkaline-extracted cellulose and commercial cellulose. The observed band characterized by O-H stretching vibrations could be correlated to the presence of aliphatic moieties in polysaccharides [32]. The C-H stretching vibration is portrayed at 2900 cm^−1^ for commercial cellulose, while a similar vibration was noticed in raw material samples at 2920–2899 cm^−1^ and extracted cellulose samples at 2917–2896 cm^−1^. These band ranges are related to the general organic component of the polysaccharides [26]. The peak at 1742 cm^−1^ and 1510 cm^−1^ corresponded to the C-O stretching vibrations of hemicellulose, and the C=C vibrations in lignin were not displayed in the observed spectra of extracted cellulose and commercial cellulose samples. The absence of absorption peaks at the aforementioned bands revealed that hemicellulose and lignin impurities were completely removed [33,34]. The O-H bending vibration owing to the moisture absorption of cellulose denotes the peaks at wavenumbers of 1636–1612 cm^−1^ for all samples. The absorption peaks at wavenumber 1058 and 897 cm^−1^ are related to the C-O-C and C-O stretching at the β-glycosidic linkages [35]. In addition, the substantial peaks that appear in the range from 1200 to 890 cm^−1^ in the cellulose samples showed increased cellulose contents after chemical treatment [36]. The findings are in line with the increase in cellulose content and decreased lignin, hemicellulose, of extracted cellulose samples compared with the raw materials, as shown in Table 1. Additionally, the AE-CCC sample contained a minute quantity of lignin and hemicellulose compared to the other raw materials and alkaline-extracted cellulose samples, as displayed in the FTIR spectra.

### 3.4. X-ray Diffraction (XRD) of Cellulose Samples Extracted without and with Alkaline Process

**Figure 3:**
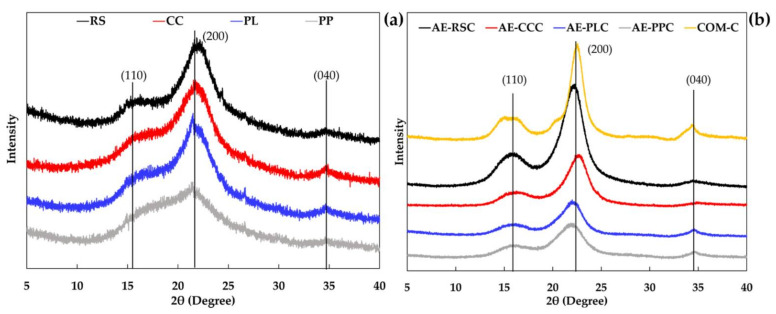
XRD spectra of cellulose samples without (**a**) and with (**b**) alkaline processing of agricultural waste materials and their comparison with the commercial cellulose. RS: rice straw; CC: corncob; PL: pineapple leaf; PP: pineapple peel; AE-RSC: alkaline extraction of rice straw cellulose; AE-CCC: alkaline extraction of corncob cellulose; AE-PLC: alkaline extraction of pineapple leaf cellulose; AE-PPC: alkaline extraction of pineapple peels cellulose; and COM-C: commercial cellulose.

The XRD spectra of crude cellulose (without alkaline treatment) and alkaline-extracted cellulose samples of different agricultural by-products are provided in Figure 3, and the calculated crystallinity index (CI) is shown in Table 2. The major diffraction peaks were observed at 2θ = 16°, 22.5°, and 34.5° for all raw material and extracted cellulose samples, which corresponds to the crystallographic planes of (110), (200) and (040), indicating the cellulose-I structure o [37]. These results revealed that all raw materials and cellulose samples exhibited a crystalline structure denoted as cellulose-I. The similar patterns across the various raw material sources and alkaline-extracted cellulose samples suggested that the type of raw materials and cellulose extraction methods had no major impact on the natural cellulose-I structure. CI assessed by XRD displays the changes in the physical and mechanical properties of the materials. The CI values of RC, CC, PL, and PP samples were 33.57%, 21.40%, 20.73% and 2.38%, respectively (Table 2). Raw material samples are composed of amorphous and crystalline regions in which hemicellulose corresponds to the amorphous part, and lignin conjugated to cellulose includes both amorphous and crystalline regions in biopolymers [38,39]. After the bleaching and alkaline-extraction process, the CI of cellulose samples was higher than the raw material employed for comparison without alkaline treatment. This result was in line with the higher crystallinity peak attained in the extracted cellulose samples from various agricultural waste materials [40,41]. Among the extracted cellulose samples, AE-CCC presented the highest in CI (69.45%), followed by AE-RSC (66.10%), AE-PLC (51.75%), and AE-PPC (44.58%). However, the COM-C sample exhibited the highest crystallinity (80.14%) than the raw and alkaline-treated samples.

### 3.5. Thermogravimetric Analysis (TGA) and Derivative Thermogravimetry (DTG) Curves of Extracted Cellulose with Alkaline Process

**Figure 4:**
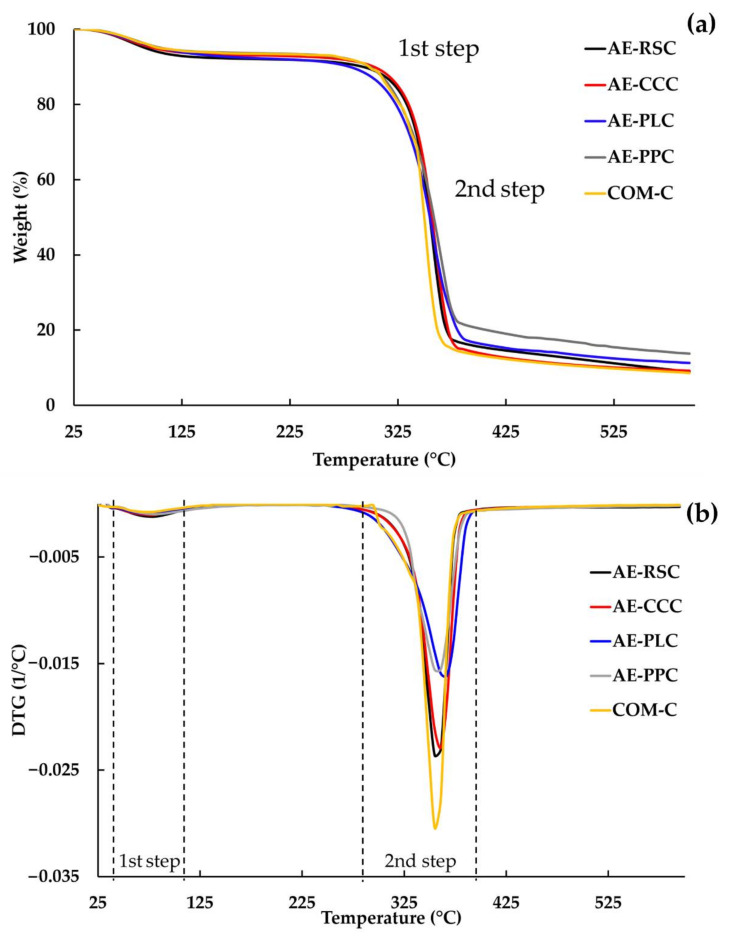
(**a**) Thermogravimetric analysis (TGA), and (**b**) derivative thermogravimetry (DTG) curves of cellulose samples extracted with alkaline processing of agricultural waste materials and their comparison with the commercial cellulose. AE-RSC: alkaline extraction of rice straw cellulose; AE-CCC: alkaline extraction of corncob cellulose; AE-PLC: alkaline extraction of pineapple leaves’ cellulose; AE-PPC: alkaline extraction of pineapple peels’ cellulose; and COM-C: commercial cellulose.

Thermal stability determination is critical for applications that require a higher processing temperature, most notably in the production of bio-composites. The TGA and DTG curves of cellulose extracted from different sources, compared with commercial cellulose, are presented in Figure 4. The degradation temperature of lignin, hemicellulose, and cellulose are within the temperature ranges of 160–900, 220–315 and 315–400 °C, respectively [42]. From Figure 4a, it can be seen that all extracted cellulose samples and COM-C sample exhibited single-step degradation in the temperature range of 320–370 °C, which indicates that the degradation was mainly due to one component. This is supported by the results of DTG, which showed single peaks for all samples. The initial weight loss of 5–8% in all cellulose samples was observed between the temperature range from 60 to 125 °C. These slight weight losses in cellulose samples are related to the evaporation of volatile chemicals and adhered water droplets [43]. Then, most of the degradation occurred at 320 to 370 °C, owing to the decomposition of cellulose. The peak degradation temperatures (T_max_) of the AE-RSC, AE-CCC, AE-PLC, AE-PPC, and COM-C samples were obtained at 360, 364, 357, 358, and 357 °C, respectively (Table 2). Of these, the AE-CCC sample indicated a higher degree of T_max_, possibly due to complex molecular arrangements. Additionally, the higher thermal degradation in alkaline-treated samples might be correlated with the removal of hemicellulose and lignin, as evidenced by the absence of peaks in Figure 4. Moreover, the absence of lignin and hemicellulose degradation curves in the TGA of samples might be due to the removal of impurities in cellulose [44]. The final residues, remaining at 600 °C after thermal degradation of all the samples, depended on the type of raw material. The AE-PPC sample marked the highest final residua (18.40%), followed by AE-PLC (15.44%), AE-RSC (15.24%), AE-CCC (13.37%), and COM-C (12.55%), respectively. The low value of char residua is indicated the high purity of cellulose [45]. The high residual was mainly due to the presence of flame retardation compounds, which result in char formation [46]. This finding is in line with the chemical composition from Table 1. Variations in chemical structure and crystallinity may influence the variances in final residues among the cellulose. The thermal degradation behavior of extracted cellulose was in line with the previous study of cellulose isolated from crop harvesting waste materials [27].

## 4. Conclusions

Corncob, rice straw, Phulae pineapple leaves and peel waste valorization with prior bleaching and alkaline treatment yielded higher cellulose contents compared to the crude waste samples without any treatment. The AE-CCC sample showed lowest amounts of hemicellulose and lignin, followed by the highest extraction yield (38.18%) and α-cellulose (82.69%). Microstructure displayed the fibrous cellulose structure in AE-RSC and AE-PLC samples and a flaky structure was visualized in AE-CCC and AE-PPC samples that resembled the COM-C sample. The FTIR confirmed that, after bleaching and alkaline treatment, the main component in samples is cellulose. The XRD of all alkaline-treated samples presented an increased CI compared to the untreated raw material. The highest CI value was attained in AE-CCC, probably due to the sufficient elimination of hemicellulose and lignin, which are known as amorphous components. TGA and DTG results reported the excellent thermal stability of all the alkaline-processed cellulose samples in which AE-CCC showed the highest stability at T_onset_ (334.64 °C), T_max_ (364.67 °C) with residue at 600 °C (13.37%).

Therefore, this cellulose, extracted via alkaline extraction generated from the agricultural wastes, should be further investigated at the nano-scale level for the development of intelligent food packaging material, subject to the assessment of legal standards to claim it as an eco-friendly substitute to plastic packaging in the quality preservation of perishable food products. This may also eradicate the problem of burning agricultural wastes in crop fields, which causes a high rise in particulate matter of up to 2.5 due to the amount of smoke leading to environmental pollution and health concerns in the local community of Chiang Rai, Thailand. Moreover, further work should be conducted to demonstrate the waste utilization process as a vital asset to the economy of local farmers.

## Figures and Tables

**Table 1:** Physico-chemical compositions of cellulose extracted from agricultural waste without and with alkaline extraction process.

Samples	Yield (% *w*/*w*)	Ash (% *w*/*w*)	Lignin (% *w*/*w*)	Hemicellulose (% *w*/*w*)	α-Cellulose (% *w*/*w*)
RS	-	9.96 ± 0.14 ^a^	21.63 ± 0.71 ^a^	31.01 ± 0.99 ^b^	45.45 ± 1.35 ^c^
CC	-	3.17 ± 0.13 ^d^	14.93 ± 0.36 ^c^	27.78 ± 1.65 ^c^	45.81 ± 0.61 ^c^
PL	-	6.35 ± 0.20 ^b^	27.25 ± 0.43 ^b^	31.05 ± 1.46 ^b^	35.35 ± 1.01 ^d^
PP	-	4.79 ± 0.21 ^c^	12.70 ± 1.45 ^d^	44.15 ± 2.63 ^a^	33.18 ± 1.47 ^e^
AE-RSC	32.26 ± 1.34 ^b^	0.68 ± 0.10 ^e^	0.89 ± 0.03 ^e^	13.51 ± 0.45 ^d^	79.19 ± 0.69 ^b^
AE-CCC	38.18 ± 0.66 ^a^	0.42 ± 0.03 ^f^	0.58 ± 0.06 ^e^	9.61 ± 0.75 ^e^	82.69 ± 1.10 ^a^
AE-PLC	16.60 ± 1.42 ^c^	0.87 ± 0.03 ^e^	0.68 ± 0.10 ^e^	13.24 ± 0.21 ^d^	78.64 ± 0.47 ^b^
AE-PPC	9.05 ± 0.07 ^d^	0.77 ± 0.03 ^e^	0.27 ± 0.04 ^e^	14.60 ± 1.85 ^d^	78.07 ± 1.44 ^b^

Values are presented as mean ± standard deviation (*n* = 3). Different superscripts (^a–f^) in each column are significantly different (*p* < 0.05). RS: rice straw; CC: corncob; PL: pineapple leaf; PP: pineapple peel; AE-RSC: Alkaline extraction of rice straw cellulose; AE-CCC: Alkaline extraction of corncob cellulose; AE-PLC: Alkaline extraction of pineapple leaves cellulose; AE-PPC: Alkaline extraction of pineapple peels cellulose.

**Table 2:** Crystallinity index (CI) and thermal degradation temperature of cellulose samples extracted without and with the alkaline process.

Samples	CI (%)	T_onset_ (°C)	T_max_ (°C)	Residue at 600 °C (%)
RS	33.57	-	-	-
CC	21.40	-	-	-
PL	20.73	-	-	-
PP	2.38	-	-	-
AE-RSC	66.10	330.53	360.00	15.24
AE-CCC	69.45	334.64	364.67	13.37
AE-PLC	51.75	331.37	358.00	15.44
AE-PPC	44.58	323.07	357.67	18.40
COM-C	80.14	321.10	357.00	12.55

Values presented the CI of cellulose samples extracted without and with alkaline processing of agricultural waste materials and their comparison with the commercial cellulose. Values presented the onset decomposition temperature (T_onset_), peak decomposition temperature (T_max_), and percent residue (600 °C) of cellulose samples extracted with alkaline processing of agricultural waste materials and their comparison with the commercial cellulose. RS: rice straw; CC: corncob; PL: pineapple leaves; PP: pineapple peel; AE-RSC: alkaline extraction of rice straw cellulose; AE-CCC: alkaline extraction of corncob cellulose; AE-PLC: alkaline extraction of pineapple leaves cellulose; AE-PPC: alkaline extraction of pineapple peels cellulose; and COM-C: commercial cellulose.

## Data Availability

The data presented in this study are available on request from the corresponding author.
